# Supplementary material for: The causal role of circulating inflammatory markers in osteoporosis: a bidirectional Mendelian randomized study
Source: Front Immunol. 2024 Jul 18;15:1412298. doi: 10.3389/fimmu.2024.1412298 (PMC11291241; doi:10.3389/fimmu.2024.1412298)
Supplement: Supplementary file 1 [file DataSheet_1.zip › supplementary material/CIM-OSTEOPOROSIS /GCST90274833.csv_leave_one_out.pdf]

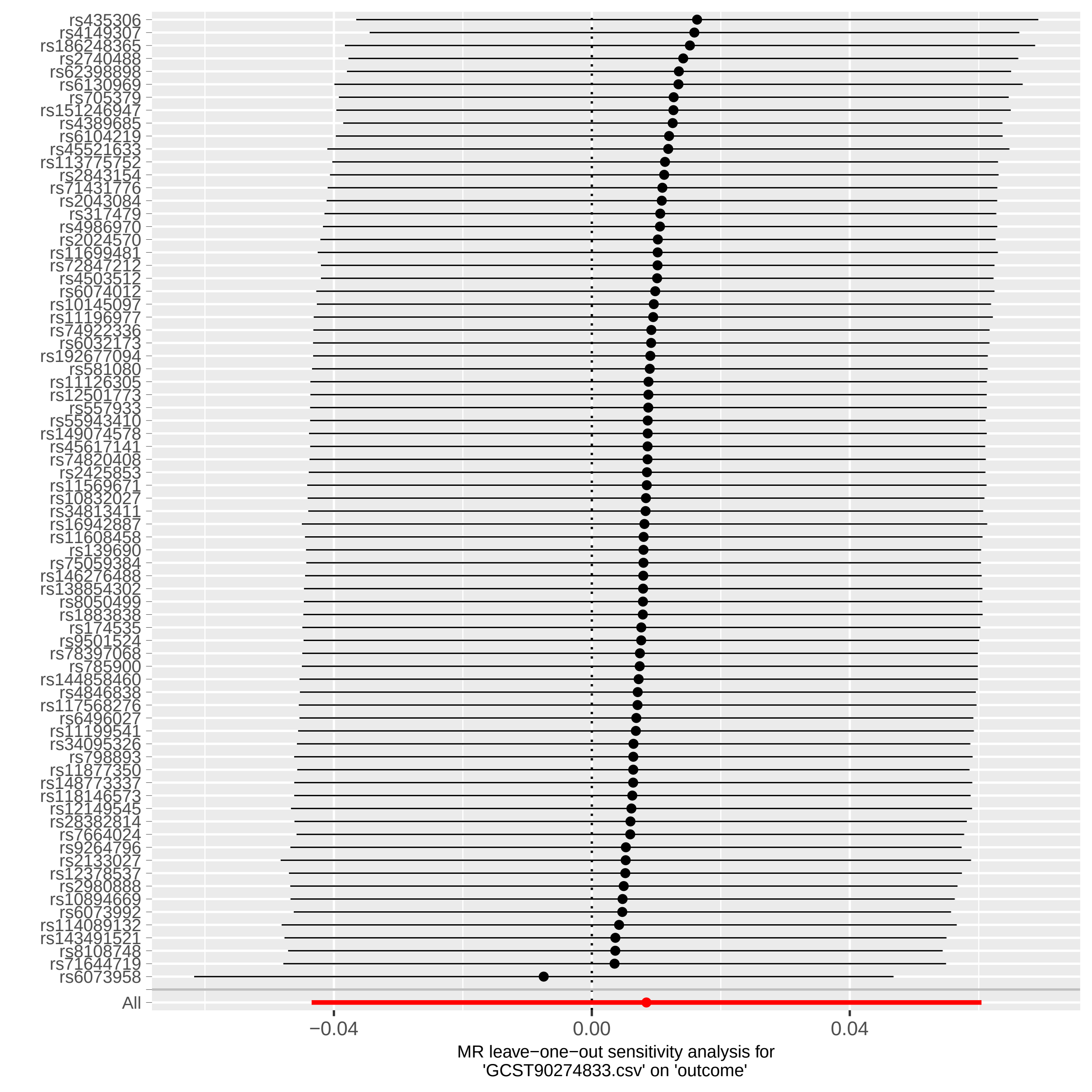

rs435306
rs4149307
rs186248365
rs2740488
rs62398898
rs6130969
rs705379
rs151246947
rs4389685
rs6104219
rs45521633
rs113775752
rs2843154
rs71431776
rs2043084
rs317479
rs4986970
rs2024570
rs11699481
rs72847212
rs4503512
rs6074012
rs10145097
rs11196977
rs74922336
rs6032173
rs192677094
rs581080
rs11126305
rs12501773
rs557933
rs55943410
rs149074578
rs45617141
rs74820408
rs2425853
rs11569671
rs10832027
rs34813411
rs16942887
rs11608458
rs139690
rs75059384
rs146276488
rs138854302
rs8050499
rs1883838
rs174535
rs9501524
rs78397068
rs785900
rs144858460
rs4846838
rs117568276
rs6496027
rs11199541
rs34095326
rs798893
rs11877350
rs148773337
rs118146573
rs12149545
rs28382814
rs7664024
rs9264796
rs2133027
rs12378537
rs2980888
rs10894669
rs6073992
rs114089132
rs143491521
rs8108748
rs71644719
rs6073958
All
−0.04
0.00
0.04
MR leave−one−out sensitivity analysis for
'GCST90274833.csv' on 'outcome'
